# Oral peripheral nerve sheath tumors: A clinicopathological and immunohistochemical study of 32 cases in a Brazilian population

**DOI:** 10.4317/jced.54338

**Published:** 2017-12-01

**Authors:** Talita Franco, Silas-Antonio-Juvencio de Freitas Filho, Laís-Borges Muniz, Paulo-Rogério de Faria, Adriano-Mota Loyola, Sérgio-Vitorino Cardoso

**Affiliations:** 1 DDS, MSc, Area of Pathology, School of Dentistry, Federal University of Uberlândia, Uberlândia, MG, Brazil; 2 DDS, MSc, PhD, Professor, Department of Morphology, Institute of Biomedical Sciences, Federal University of Uberlândia, Uberlândia, MG, Brazil; 3 DDS, MSc, PhD, Professor, Area of Pathology, School of Dentistry, Federal University of Uberlândia, Uberlândia, MG, Brazil

## Abstract

**Background:**

Oral peripheral nerve sheath tumors (OPNSTs) are reactive or neoplastic diseases that develop from proliferation of the nerve itself or their limiting sheaths. Here we describe the clinicopathologic data of OPNSTs observed in a sample of the Brazilian population and evaluate the expression of molecules associated with neural biology to determine their usefulness in the diagnosis.

**Material and Methods:**

Descriptive study of cases diagnosed as OPNSTs, from the Pathology Laboratory at the School of Dentistry/ Federal University of Uberlandia, followed by an immunohistochemical study of S-100, CD57, neurofilament protein (NFP) and epithelial membrane antigen (EMA).

**Results:**

OPNSTs comprised 0.27% of all biopsies. There were eight patients with neurofibromas, eight with traumatic neuromas, seven with schwannomas, five with granular cell tumor (GCT), and four with palisaded encapsulated neuromas (PEN). Women were more frequently affected (60.6% of the cases). Tongue and lips prevailed as the most frequent sites. S-100 was reactive in 100% of the cases. Neural fibers evidenced by CD57 reactivity of their Schwann cells were always nested in bundles within neurofibromas and GCT, absent within schwannomas and dispersed within PEN. Reactivity for NFP was limited to axons and then followed the same pattern of CD57, though much less evident. Reactivity for EMA was observed in the capsular tissues and perineurium of nerve fascicles, and absent in parenchymal cells of GCT.

**Conclusions:**

This study showed that OPNSTs are rare, widely benign and often found in tongue and lips. OPNSTs evolve from a common origin to distinct histological patterns, with eventual overlapping in their clinical and morphologic features. The arrangement of reactive residual neural fibers for CD57 can be a useful staining in the differential diagnosis of OPNSTs.

** Key words:**Peripheral nerve sheath tumors. Oral cavity. Differential diagnosis. Immunohistochemistry. CD57 antigens.

## Introduction

Nerves are the main constituent of the peripheral neural system and are composed of single or multiple neural fibers. Each neural fiber is composed by an axon surrounded by Schwann cells and is encased by an endoneurial space composed by a thin basal lamina produced by Schwann cells and eventual endoneurial fibroblasts. Multiple neural fibers are grouped in fascicles by an external layer of dense connective tissue named perineurium, and many fascicles can be joined together within a thick mesh of epifascicular epineural connective tissue that also forms a dense membrane known as epineural epineurium (1,2).

Among several diseases that can affect the nerves, peripheral nerve sheath tumors (PNSTs) are reactive or neoplastic diseases that develop from proliferation of the nerve itself (axons and Schwann cells) or their limiting sheaths (2,3). PNSTs have been classified according to their cellular composition and organization. The list of PNSTs is extensive, but neurofibromas, schwannomas, traumatic neuromas, palisaded encapsulated neuromas (PEN), granular cell tumors (GCT), nerve sheath myxomas, and perineuriomas, in addition to malignant peripheral nerve sheath tumors (MPNSTs), have been mentioned as the most common PNSTs (2-4).

Many of these lesions affect the head and neck, most of them in the face and scalp, and the oral cavity has been regarded as a less frequent site for these tumors (5). There are few comparative studies on the epidemiology and pathological diagnosis of oral peripheral nerve sheath tumors (OPNSTs) (6-10). This is particularly important in face of the eventual overlapping of microscopic features among some of these lesions, and then immunohistochemical analysis has been regarded as relevant for a conclusive diagnosis (3,4). However, substantial doubt remains. For example, there is great variation in the proportional frequency of PEN (6-9)

The aim of this paper was to describe the experience of a Pathology Department with the diagnosis of OPNSTs, as well as to evaluate the expression in these lesions of molecules associated with neural biology to determine their usefulness in the differential diagnosis.

## Material and Methods

This study was approved by the Human Research Ethics Committee of the Federal University of Uberlandia (protocol nº. 410/11).

This work included all of the lesions diagnosed as OPNTSs from 1978 to 2011 at the Oral Pathology Service of the Dental School of the Federal University of Uberlandia. The dental and medical files of the patients were reviewed to obtain demographic and clinical information.

Immunohistochemistry was performed in formalin-fixed, paraffin-embedded archival samples to detect S-100, CD57, neurofilament protein (NFP), and epithelial membrane antigens. The details of this procedure are listed in  Table 1. Amplification was performed with a streptavidin-biotin-peroxidase kit (LSAB+, Dako, USA), with staining development with diaminobenzidine (Dako, USA) and hematoxylin counterstaining. The immunohistochemical results were evaluated according to the presence and cellular allocation of reactivity.

**Table 1:**
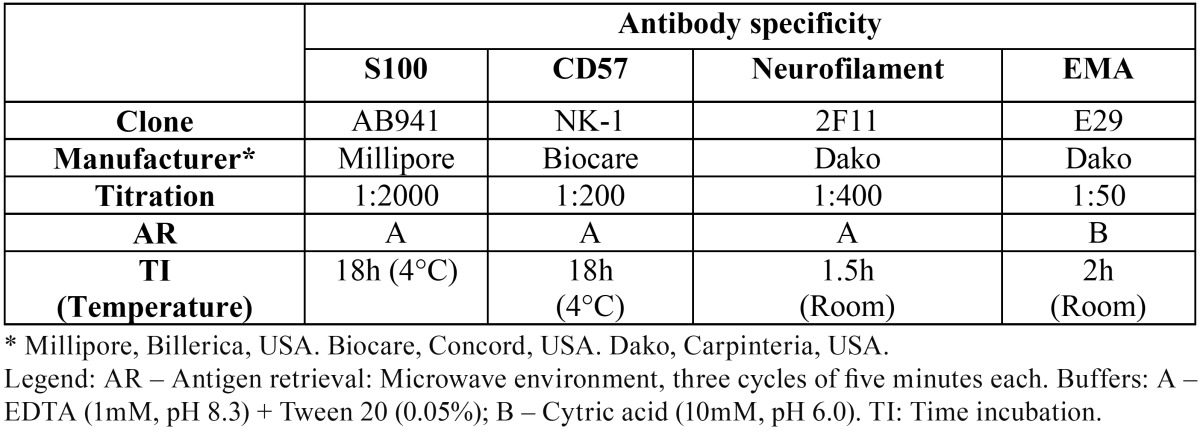
Antibodies and procedures used for immunohistochemistry.

## Results

Thirty-two patients with OPNSTs were retrieved. These lesions comprised eight patients with neurofibromas – one of which presented neurofibromatosis and had two lesions biopsied, eight with traumatic neuromas, seven with schwannomas, five with granular cell tumors – one of which presented multiple and four with palisaded encapsulated neuromas. These 34 lesions represented 0.27% of nearly 13,000 specimens examined in the same period. The individual data for these cases are presented in  Table 2.

**Table 2:**
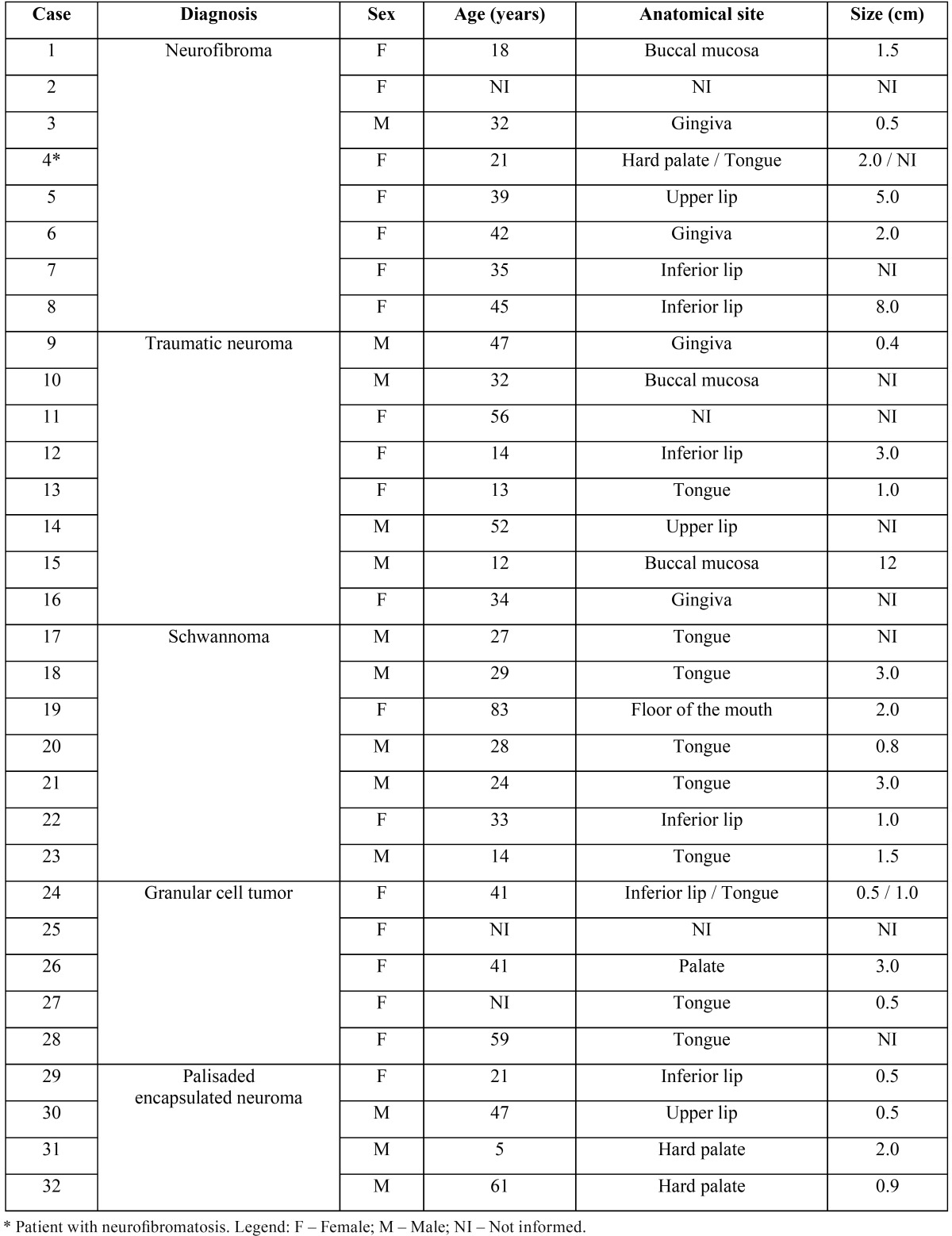
Clinical data of 32 patients with oral peripheral nerve sheath tumors (1978-2011).

These OPNSTs more frequently affected women (60.6% of the cases). All the GCTs were found in women, who also predominated among patients with neurofibroma (87.5% of cases). In contrast, PEN and schwannoma were more frequently found in men (75.0% and 71.4%, respectively). Most of these OPNSTs were diagnosed in adults (21 cases, 65.6%). Patients with traumatic neuromas (50.0% of the cases) predominated among those up to 20 years of age.

The tongue and lips predominated as the most frequent sites for OPNSTs, with each location accounting for 31.0% of the cases. Areas with masticatory mucosa (palate and gingiva) were also frequently affected (23.8%). Schwannomas were not found in areas with masticatory mucosa, and PENs were not found in the tongue. The lesions were usually small, but tumors of 3cm or more were observed for schwannomas, traumatic neuromas, neurofibromas, and GCTs.

**Figure 1:**
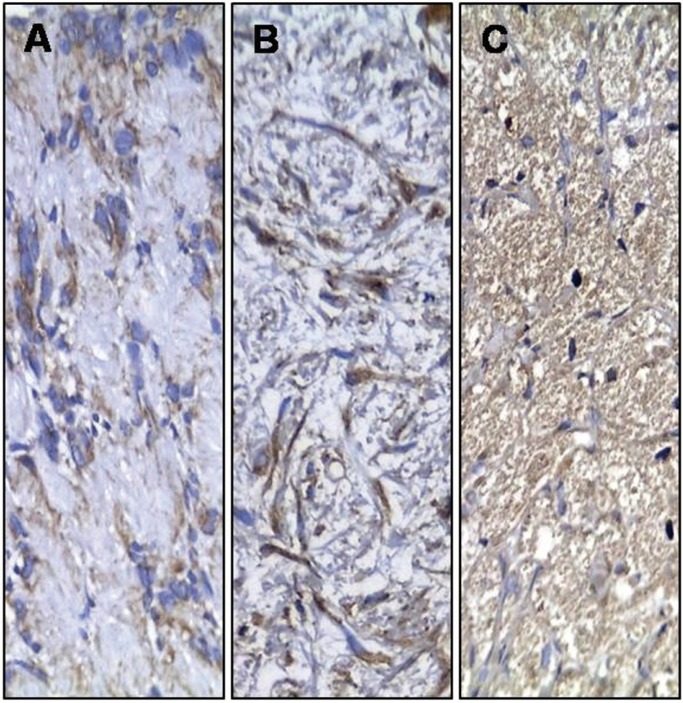
Immunohistochemical reactivity for S-100 protein in schwannoma (A), neurofibroma (B) and granular cell tumor (C) [Streptavidin-biotin-peroxidase method; original magnification (A-C) x200].

**Figure 2:**
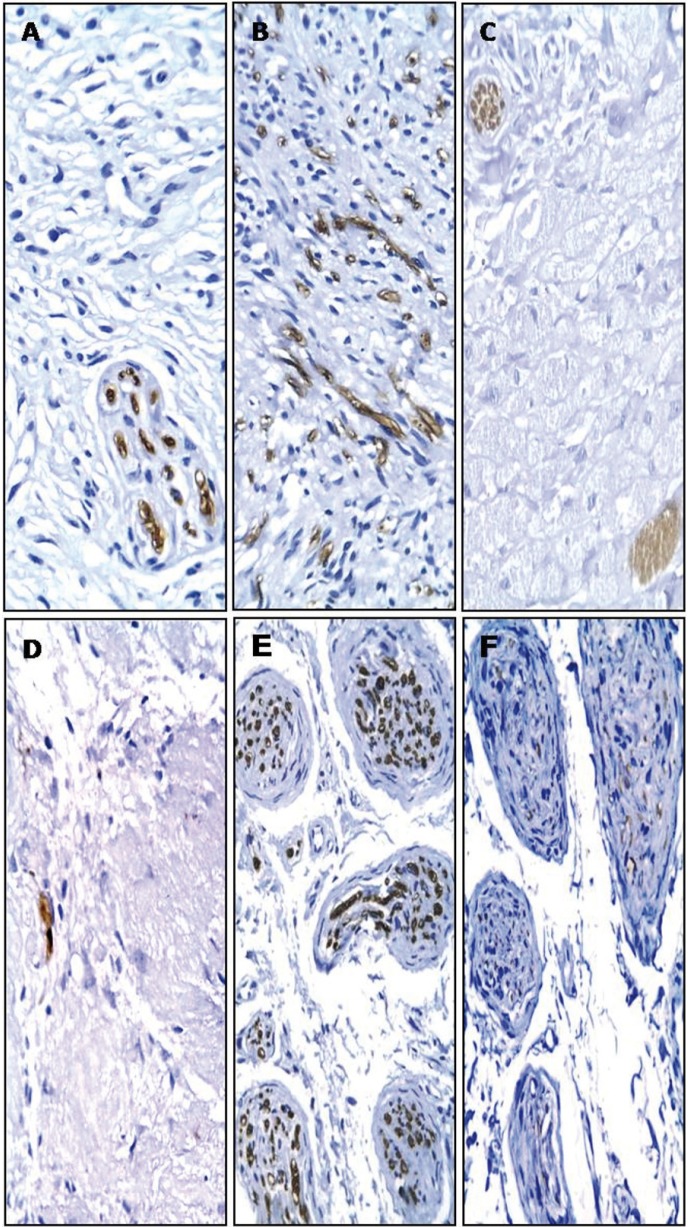
Immunohistochemical reactivity for CD57 antigen in nerve fascicles of neurofibroma (A), diffuse and high quantity of positive cells in PEN (B), nerve fascicles and rare positive cells in GCTs (C), infrequent in neoplastic cells of schwannoma (Antoni B tissue) (D) and positive Schwann cells in traumatic neuroma (E). Neurofilament protein staining was limited to axons in neuroma traumatic (F) [Streptavidin-biotin-peroxidase method; original magnifications (A-F) x200].

**Figure 3:**
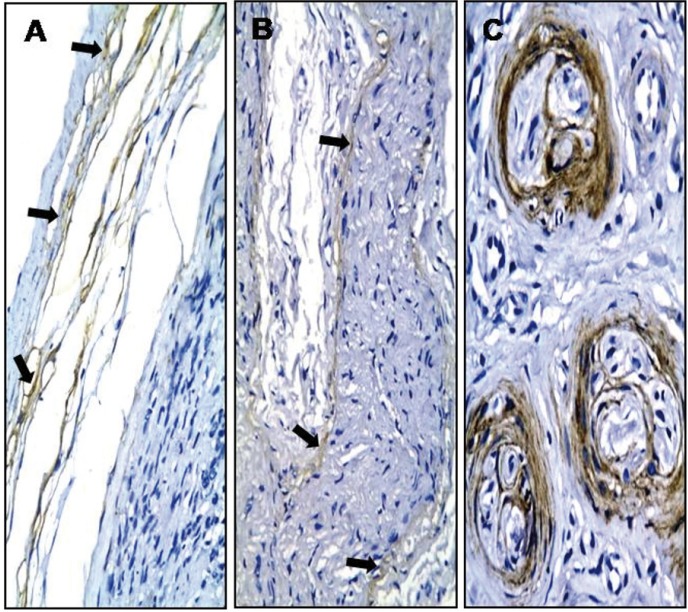
Immunohistochemical reactivity for epithelial membrane antigen (EMA) in capsular tissues of schwannoma (A) (arrows), in capsule surrounding lobules of palisaded encapsulated neuroma (B) (arrows) and in the perineurium of traumatic neuroma (C) [Streptavidin-biotin-peroxidase method; original magnifications (A, B) x200, (C) x400].

Representative pictures of the immunohistochemical reactivity are presented in Figures 1 to 3. Except for three cases (one neurofibroma, one traumatic neuroma, and one GCT) that were not submitted to immunohistochemistry due to an insufficient amount of archival tissue, most of the cells of all of the current OPNSTs were diffusely reactive for the S-100 antigen (Fig. 1). It was always observed in the cytoplasm, with a perinuclear concentration in a case of schwannomas (Fig. 1A). Nuclear reactivity for S-100 was also present in 43% of neurofibromas (Fig. 1B), 50% of PENs, 29% of schwannomas and 60% of GCTs (Fig. 1C). Reactivity for CD57 evidencing nerve fascicles were found within neurofibromas (Fig. 2A), diffuse and high quantity of positive cells in PENs (Fig. 2B), nerve fascicles and rare cells in GCTs (< 5%) (Fig. 2C), and infrequent in neoplastic cells of schwannomas (Fig. 2D). In addition, intense CD57 reactivity was also evident in the Schwann cells of traumatic neuromas (Fig. 2E). The immunohistochemical reactivity for NFP was limited to axons, which were distributed in the same patterns described for CD57, but it was less evident due to the reduced axonal thickness (Fig. 2F). Reactivity for epithelial membrane antigen (EMA) was observed in the capsular tissue of schwannomas (Fig. 3A), less intensely in the perineurial capsule surrounding lobules of PENs (Fig. 3B), and in the perineurial cells of hyperplastic nerve fascicles in traumatic neuromas (Fig. 3C).

## Discussion

Tumors of neural origin are rare in soft tissue and much less common in the mouth: as observed here PNSTs represent less than 0.5% of biopsies performed in the oral mucosa (9,11). Despite numerous papers on single histological types of OPNSTs, only a few comparative series are available in the international literature (6,7,9,10,12). These papers have not observed the same list of lesions, and a discrepancy is also observed in comprehensive publications, where lesions recognized as PNSTs (neurofibroma, schwannoma, lipomatosis of the nerve, palisaded encapsulated neuroma, perineurioma, traumatic neuroma, nerve sheath myxoma, granular cell tumor, and malignant peripheral nerve sheath tumor) are differently grouped (3,4,6,7,9,10,12).

 Table 3 shows the relative frequency of each PNSTs of the oral mucosa as reported in the available comparative papers (PubMed). Neurofibroma has been consistently described as the first or second most frequent of these lesions. It is followed by traumatic neuromas and schwannomas, but their relative frequency varies widely in the same way as the less frequently found PEN, GCT, and MPNSTs. This inconsistency can be partially credited to selection bias since the paper of Jordan *et al.* (7) was restricted to spindle cell tumors and reported the smallest frequency of traumatic neuromas and no GCTs.

**Table 3:**
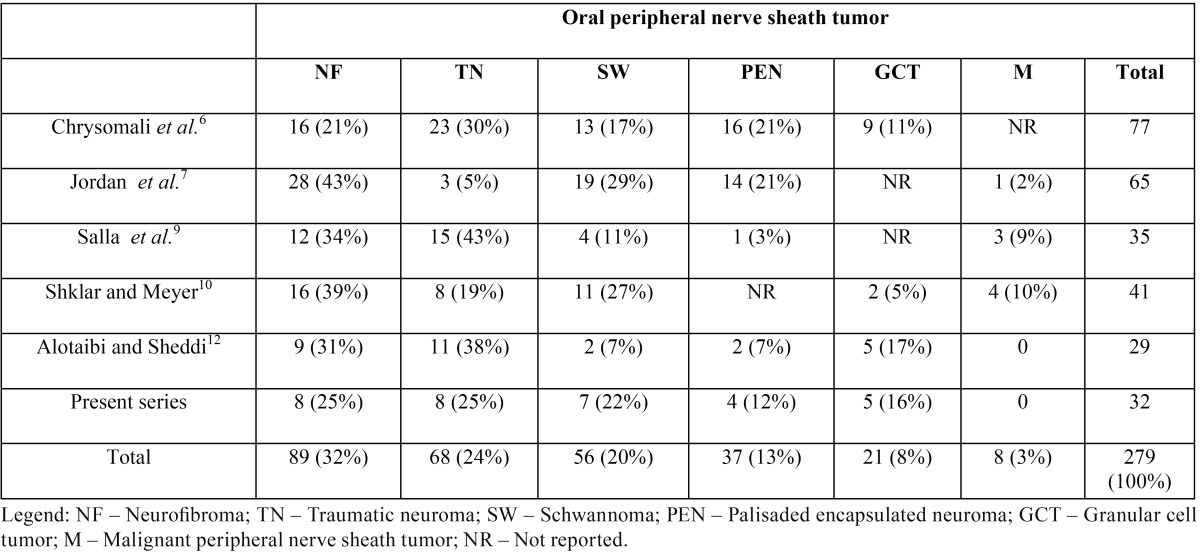
Proportional frequency of peripheral nerve sheath tumors of the oral mucosa in different case series available in the international literature (PUBMED).

In addition, the study of Chrysomaly *et al.* (6) was limited to benign PNSTs and, therefore, did not address malignant cases; the paper by Shklar and Meyer (10) was published before the description of PEN; Salla *et al.* (9) apparently did not consider GCT to be a PNSTs; melanotic neuroectodermal tumor of infancy reported by Alotaibi and Al Sheddi (12) is not reported in others studies (6,7,9,10). Katz and McAlpin (13) found only one case involving oral mucosa of 32 patients with face and neurogenic neoplasms, who had a buccal carcinoma and a “nodal metastatic mass”, which was surprisingly a schwannoma.

There are recognized diagnostic challenges with some OPNSTs that can also be held responsible for their unclear relative frequency. This is particularly true for tumors composed of spindle cells, especially with respect to the differentiation between schwannomas and PENs and less notably between the latter and neurofibromas (6). Immunohistochemistry is a common aid in the diagnostic study of spindle cell tumors. Reactivity for S-100 antigen has been highlighted as the most relevant finding to corroborate neurogenic origin, including the GCTs (6,7). In the present study, a minor variation was observed in the subcellular compartmentalization of S-100 reactivity, with schwannomas showing less frequent nuclear positivity than neurofibromas and PENs. Although Chrysomali, *et al.* (6) did not explore this finding, their second figure also presents noticeable differences between schwannomas and PENs regarding nuclear reactivity for S-100.

Since the presence and allocation of axons vary among different OPNSTs, immunohistochemical detection of CD57 is an important diagnostic tool for these lesions. The low expression of CD57 in schwannomas (Fig. 2D) is substantial in distinguishing this lesion from the other spindle cell OPNSTs. Reactive cells were diffusely observed in PENs (Fig. 2B), while these structures were usually arranged in bundles in neurofibromas (6,14). Furthermore, this finding was also reported by Chrysomali, *et al.* (6), but these authors did not detail the distribution of neural fibers in PENs and neurofibromas. We observed here that the fascicle arrangement of axons (bundles) was restricted to the latter in the same way as the first figure of their paper.

Koutlas and Sheithauer (14) studied immunohistochemical reactivity for NFP in PEN and also indicated that only single axons were found in these lesions. In the present study, reactivity for CD57 in the thick myelinated Schwann cells was much more evident than the positivity for NFP in the thin axons. Of interest was also the absence of reactivity for EMA within OPNSTs with spindle cell morphology, eliminating the possibility of a perineurioma altogether with the S-100 positivity observed in all of the lesions (3,15).

In the present study, nerve bundles reactive for CD57 and neurofilament protein were consistently observed within GCTs. This contrasts with previous papers (16,17) reporting that this close relationship between GCT and nerves was uncommon. However, those studies based their impression only on the morphologic study of hematoxylin and eosin-stained sections. GCTs were also positive for S-100 antigen and exhibited rare parenchymal cells reactive for CD57. This positivity for CD57 in some parenchymal cells of GCTs was also observed by Chrysomali *et al.* (6), suggesting myelin production by some tumors cells. All of these findings support derivation from peripheral nerves rather than muscle fibers in the same way as calretinin and neuron-specific enolase positivity and lack of reactivity for muscle-specific antigens as reported in previous papers (1,16,17,18). In addition, the negative immunoreactivity was observed for EMA in parenchymal cells GCTs, similarly to other studies (6,18).

In the oral mucosa, PNSTs are typically diagnosed in adults. There is no clear sex aggregation when evaluating the entire group of these lesions, although a previous studies observed a remarkable prevalence of affected women (6,9). Most patients with neuro-fibromas, traumatic neuromas, or GCTs are women, (6,8,9,18) while men predominate among those with PEN (6,14). Still, the high prevalence of GCTs in women does not appear to be associated with hormonal factors (18). Areas covered by masticatory mucosa have been described as the most frequently affected by PNSTs, and most neurofibromas and PENs are found in such locations (6,9). Schwannomas are found in several locations, but are frequent in the lips (6,19), GCTs in the tongue (6,12,18,20) and traumatic neuromas in the tongue and lips (6,10,12,21).

A neuroma is a tumor originated in areas of nerve injury as fractures or even after some surgical procedure, clinically presenting pain, and microscopically numerous nerve fibers randomly arranged (10). Neuroma traumatic is found the region with reported trauma, while the etiologic factor for PEN remains uncertain (22). Argeniy, Cruz and Bromley (22) performed a detailed study comparing PEN and traumatic neuroma and noted structural and histochemical differences between them, also suggested different histogenesis for the lesions.

In the current study, two patients were observed with multiple OPNSTs, one of them with neurofibromatosis. This syndrome must be investigated for patients with a diagnosis of any PNSTs of the oral mucosa, and most patients with neurofibromatosis present neurofibromas in the tongue or in areas covered by masticatory mucosa (9,23). Another of our cases presented with multiple GCTs and was not associated with a syndrome (24).

In conclusion, this study showed that OPNSTs are rare, frequently benign and found in tongue and lips. OPNSTs evolve from a common origin to distinct histological patterns, with eventual overlapping in their clinical and morphologic features; there is mounting evidence that GCT is derived from peripheral nerves, but the relative frequency of this tumor is underestimated in comparative case series. Furthermore, immunohistochemical analysis revealed that the presence and arrangement of nerve fibers as highlighted by immunohistochemical staining of CD57 antigen may be useful criteria for the differential diagnosis of the spindle cell types of OPNSTs.
